# Liraglutide Exerts Protective Effects by Downregulation of *PPARγ*, *ACSL1* and *SREBP-1c* in Huh7 Cell Culture Models of Non-Alcoholic Steatosis and Drug-Induced Steatosis

**DOI:** 10.3390/cimb44080239

**Published:** 2022-08-02

**Authors:** Tea Omanovic Kolaric, Tomislav Kizivat, Vjera Mihaljevic, Milorad Zjalic, Ines Bilic-Curcic, Lucija Kuna, Robert Smolic, Aleksandar Vcev, George Y. Wu, Martina Smolic

**Affiliations:** 1 Department of Pharmacology and Biochemistry, Faculty of Dental Medicine and Health Osijek, Crkvena 21, 31000 Osijek, Croatia; tomanovic@fdmz.hr (T.O.K.); vnincevic@fdmz.hr (V.M.); lkuna@fdmz.hr (L.K.); rsmolic@fdmz.hr (R.S.); 2 Department of Pharmacology, Faculty of Medicine Osijek, J. Huttlera 4, 31000 Osijek, Croatia; ibcurcic@mefos.hr; 3 Department of Nuclear Medicine and Oncology, Faculty of Medicine Osijek, J. Huttlera 4, 31000 Osijek, Croatia; tkizivat@mefos.hr; 4 Clinical Institute of Nuclear Medicine and Radiation Protection, University Hospital Osijek, J. Huttlera 4, 31000 Osijek, Croatia; 5 Department of Molecular Medicine and Biotechnology, Faculty of Medicine Rijeka, B. Branchetta 20, 51000 Rijeka, Croatia; milzjalic@gmail.com; 6 Department of Endocrinology, University Hospital Osijek, J. Huttlera 4, 31000 Osijek, Croatia; 7 Department of Pathophysiology and Physiology with Immunology, Faculty of Dental Medicine and Health Osijek, Crkvena 21, 31000 Osijek, Croatia; avcev@mefos.hr; 8 Department of Medicine, Division of Gastroenterology-Hepatology, University of Connecticut Health Center, 135 Dowling Way, Farmington, CT 06030, USA; wu@uchc.edu

**Keywords:** NAS, amiodarone, liraglutide, Huh7 cell line

## Abstract

(1) Background: With the aging of the population and polypharmacy encountered in the elderly, drug-induced steatosis (DIS) has become frequent cause of non-alcoholic steatosis (NAS). Indeed, NAS and DIS may co-exist, making the ability to distinguish between the entities ever more important. The aim of our study was to study cell culture models of NAS and DIS and determine the effects of liraglutide (LIRA) in those models. (2) Methods: Huh7 cells were treated with oleic acid (OA), or amiodarone (AMD) to establish models of NAS and DIS, respectively. Cells were treated with LIRA and cell viability was assessed by MTT, lipid accumulation by Oil-Red-O staining and triglyceride assay, and intracellular signals involved in hepatosteatosis were quantitated by RT-PCR. (3) Results: After exposure to various OA and AMD concentrations, those that achieved 80% of cells viabilities were used in further experiments to establish NAS and DIS models using 0.5 mM OA and 20 µM AMD, respectively. In both models, LIRA increased cell viability (*p* < 0.01). Lipid accumulation was increased in both models, with microsteatotic pattern in DIS, and macrosteatotic pattern in NAS which corresponds to greater triglyceride accumulation in latter. LIRA ameliorated these changes (*p* < 0.001), and downregulated expression of lipogenic *ACSL1*, *PPARγ*, and *SREBP-1c* pathways in the liver (*p* < 0.01) (4) Conclusions: LIRA ameliorates hepatocyte steatosis in Huh7 cell culture models of NAS and DIS.

## 1. Introduction

Drug-induced liver injury (DILI) is an emerging public health problem with continuously increasing incidence and progression to more severe stages of liver disease. Pathophysiological mechanisms have still not been fully elucidated. The Roussel Uclaf Causality Assessment Method (RUCAM) and liver biopsy may help clinicians better diagnose and differentiate DILI in clinical practice [1]. The majority of countries and regions around the world have also compiled lists of empirical DILI treatment strategies and established guidelines. DILI is currently classified into three clinical patterns based on the serum alanine transaminase (ALT) and alkaline phosphatase (ALP) ratio from the first available biochemical test: hepatocellular, cholestatic, or mixed injury (liver-damaged target cells) [2]. In addition, DILI can also manifest as acute hepatic necrosis, chronic hepatitis, fatty liver, bland cholestasis, sinusoidal obstruction syndrome, and acute liver failure [3,4]. One specific form of DILI is a DIS, which occurs in up to 27% of DILI cases [5]. DIS can present in several histopathological forms, such as macro and microvesicular steatosis, and steatohepatitis which can then progress to liver fibrosis and eventually liver cirrhosis. It is not uncommon for DIS to be associated with non-alcoholic fatty liver disease (NAFLD). NAFLD is usually associated with the development of insulin resistance and occurs as a feature of another major global health problem, the metabolic syndrome. Nevertheless, a small proportion of NAFLD cases are due to drug toxicity, or to drugs that may exacerbate pre-existing fatty liver injury [6]. Some of the drugs capable of causing DIS are glucocorticoids, AMD, methotrexate, estrogens, tamoxifen, nonsteroidal anti-inflammatory drugs (NSAIDs), acetaminophen, 5-fluorouracil, metoprolol, valproic acid, tetracycline, aspirin, ibuprofen, zidovudine, glucocorticoids, perhexiline, and propranolol.

AMD is a class II antiarrhythmic drug used to treat severe ventricular arrhythmias. It can cause numerous side effects including significant hepatotoxicity [7]. Several mechanisms responsible for this effect have been proposed including stimulation of lipogenesis by affecting the expression of genes involved in lipogenesis (peroxisome proliferator-activated receptor *γ-PPARγ*, sterol regulatory element-binding protein 1c–*SREBP-1c*, *PPARα*), disruption of mitochondrial function followed by a decrease in β-oxidation of free fatty acids (FFAs), increased accumulation of reactive oxygen species (ROS) (affecting the expression of *CPT1*), and decreased glutathione (GSH) levels [7,8,9,10,11,12]. Although discontinuation of the potentially toxic drug is generally recommended, in some cases withdrawal may also have negative effects on patient health, considering the severity of the heart disease. Therefore, a better understanding of the development of DIS is necessary to identify appropriate diagnostics and therapeutics.

To date, various agents such as antioxidants vitamin E and C, and L-carnitine have been studied as potential therapeutic options [1,7]. Numerous studies have demonstrated the beneficial role of glucagon-like peptide-1 receptor agonists (GLP-1RA) in the treatment of NAFLD [13,14,15,16,17,18,19,20]. These drugs have a broad spectrum of effects on the liver, in addition to numerous effects on other organs. GLP-1 RA (semaglutide, exenatide, liraglutide) improve hepatic insulin sensitivity, decrease inflammation and fibrosis, and reduce hepatic de novo lipogenesis and fat deposition [21]. Semaglutide and exenatide, two of the agonists, have a stronger effect on lowering ALT levels, inflammation, and weight, while liraglutide (LIRA) has a stronger effect on reducing mild steatosis and hepatocyte ballooning [15,19,22]. Interestingly, the effects of LIRA on liver fibrosis are not so clear. Authors in the LEAN study listed the short duration of the treatment as a possible cause of the lack of improvement in fibrosis stage in LIRA treated group of patients [23]. Nevertheless, other effects, such as decreasing lipid over-accumulation by upregulating autophagy, adjusting lipid metabolism by SHP1/AMPK/SREBP1 signaling pathway, decreasing apoptosis, oxidative stress of liver cells, proliferation and activation of hepatic stellate cells through RAGE/NOX2, reducing lipotoxicity-induced oxidative stress possibly by modulation of NRF2, decreasing expression of main elements involved in lipogenesis (phospho-ACC), peroxisomal β-oxidation (*ACOX1*), and lipid flux/storage (*PPARγ*), make LIRA a promising GLP-1R agonist for treatment and/or prevention of NAS [13,16,17,24,25,26]. Considering the role of the *PPARγ* and *SREBP-1c* signaling pathway in NAS and DIS development, and LIRA hepatoprotective action, analysis of these and other signaling pathways related to it (such as Acyl-CoA Synthetase Long Chain Family Member 1, *ACSL1*, which channels fatty acids from fatty acid oxidation to lipid synthesis), could help better understand their effects on hepatocytes [13,26,27]. Yasmin et al. showed that apart from *PPARγ* involvement in fatty liver changes in rats, upregulation of *C/EBPα* (CCAAT Enhancer Binding Protein α) is also responsible for steatosis progression [28].

The Huh7 cell line was selected for developing NAS and DIS cell culture models because of its availability, easy handling, unlimited propagation potential, and stable phenotype [29]. Its limitations include a lack of substantial hepatocyte function with regard to the biotransformation capacity [29]. However, Gupta et al. showed that both Huh7 and HepG2 cell lines are suitable for the development of in vitro models of fatty liver, and that GLP-1R is present in both cell lines [30].

Further studies are needed to better understand underlying mechanisms for fatty liver development, and for the introduction of LIRA or other agents into daily practice. Therefore, the aim of the current study was to use an Huh7 cell culture model of DIS and to investigate whether LIRA could also play a protective role in the DIS model as has been shown for NAS in previous studies [30,31,32,33,34].

## 2. Materials and Methods

### 2.1. Chemicals

AMD, sodium oleate, and Oil-Red-O were purchased from Sigma-Aldrich (St. Louis, MO, USA), and LIRA was purchased from Novo Nordisk (Bagsvaerd, Denmark).

### 2.2. Cell Culture

Huh7 cell line was a generous gift from Prof. George Y. Wu, University of Connecticut Health Center in Farmington, NM, USA. Huh7 cells are a human hepatocyte cell line established from a well-differentiated liver cancer. Cells were sub-cultivated in 10 cm dishes in Dulbecco’s modified Eagle’s medium (DMEM) supplemented with 10% fetal bovine serum (FBS/Thermo Fisher Scientific Cat. No. 16000036) and 1% penicillin/streptomycin solution (Thermo Fisher Scientific Inc., Waltham, MA, USA) at 37 °C in a humidified atmosphere of 5% CO_2_ *v/v* in air. Cells were passaged every 3–4 days until 75% confluence was reached.

### 2.3. NAS and DIS Cell Culture Models

To establish NAS and DIS models, cells were cultured as mentioned above and grown overnight in a 96-well plate to reach 90% confluence. Afterward, cells were maintained in DMEM without FBS, and exposed to increasing concentrations of oleic acid (0.5 mM and 1 mM) or AMD (5, 10, 20, 40, 80 µM) for 24 h up to 72 h. Experiments were performed in triplicates [9,35,36]. Two controls were used: a negative control (cells incubated only in DMEM without FBS) and a positive control (cells incubated in OA, as NAS model). The effect of drugs on cell viability was determined by an MTT colorimetric assay (Cruz chemicals, Dallas, TX, USA) on a microplate reader (iMarkTM microplate absorbance reader; Bio-Rad, Hercules, CA, USA), according to the manufacturer’s protocol and as described previously [37]. For further experiments, optimal concentrations of AMD (20 µM) and OA (0.5 mM) and a time period of 24 h, were selected because these concentrations reduced cell viability to 80% compared to the negative control, and induced steatosis for NAS and DIS models. These results were also confirmed by the Erythrosin B color exclusion test and cell counting with a Neubauer hemocytometer (data not shown).

### 2.4. Measurement of the Hepatoprotective Effect of LIRA in NAS and DIS Cell Culture Models

LIRA was prepared in three different final concentrations (5 nM, 10 nM, 20 nM). LIRA was then added as a co-treatment to the above-mentioned NAS and DIS models. In short, first, the cells were grown overnight in 96 well and 6- well plates to reach 90% confluence. The cell subgroups for the determination of the hepatoprotective effect of LIRA were designed as follows: Huh7 cells grown in DMEM as a negative control; Huh7 cells treated with increasing concentrations of LIRA (5, 10, and 20 nM); cells treated with OA as a positive control; Huh7 cells treated with OA and increasing concentrations of LIRA; Huh7 cells treated with AMD; Huh7 cells treated with AMD and increasing concentrations of LIRA. On the second day of the experiment, determinations of cell survival and viability were performed by MTT colorimetric assay and Erythrosin B color exclusion test as previously described. Results are shown as a percentage relative to the negative control of at least three biological replicates.

### 2.5. Visualization of Fat Accumulation

According to the above-described protocol, cells were incubated and treated for 24 h in 24-well plates with cover slides previously prepared following the protocol by Zjalic et al. [38] and coated with poly-D-Lysine (Sigma Aldrich, St. Louis, MO, USA). At the end of the experiment, cells were fixed with formaldehyde for 30 min at +4 °C. Afterwards, cells were washed 2x with PBS. Neutral lipids were stained using Oil-Red-O dye (ChemCruz, Huissen, The Netherlands). Fixed cells were stained with a working Oil-Red-O solution for 30 min. Oil-Red-O 0.5% stock solution was dissolved in isopropanol, and the working solution was prepared as 60% Oil-Red-O stock solution with 40% water. Fixed cells were stained with a working Oil-Red-O solution for 30 min. After rinsing two times with phosphate buffered saline (PBS), cells were mounted in the fluorescent mounting medium with 4′,6′-diamidino-2-phenylindole (DAPI) (Abcam, Cambridge, UK). The cells were visualized using a microscope (Axioskop 2 MOT Inverted microscope, Carl Zeiss, Göttingen Germany) with an Olympus D70 camera, controlled through the computer program DP Manager 1.2.1.107 and DP Controller 1.2.1.108. ImageJ-Fiji software was used to count cell nuclei and measure integrated density relative to the cell count. Results are shown as a percentage relative to the negative control of at least three biological replicates.

### 2.6. Measurement of Triglyceride Levels

After treatment according to the above-described protocol, the accumulation of triglycerides in 12 subgroups of cells was measured by triglyceride GPO—PAP method (glycerol 3 phosphate oxidase—4-Amino-antipyrine, Greiner Diagnostic, Bahlingen, Germany). Triglyceride standards were used in order to quantify absolute concentration of triglycerides. Results were expressed as absolute values in mg/dl.

### 2.7. Total RNA Isolation and Reverse Transcription Polymerase Chain Reaction (RT-PCR) Analysis

To evaluate the expression of various genes (*ACSL1*, *C/EBPα*, *PPARγ*, *SREBP-1c*), total RNA from cells was isolated on the third day of experiment using TRI Reagent (T9424, Sigma Aldrich, St. Louis, MS, USA) according to the manufacturer’s protocol. cDNA strand was synthesized using a commercially available kit (High-Capacity cDNA Reverse Transcription Kit, Applied Biosystems, Thermo Fisher Scientific Inc., Waltham, MA, USA) according to the manufacturer’s instructions. A commercially available kit (Taq PCR Core Kit, Qiagen, Hilden, Germany) was used to amplify the obtained cDNA using a DNA Engine^®^ Thermal Cycler (Bio-Rad, Hercules, CA, USA, SAD). PCR conditions are listed in Table 1. The synthesized cDNA was amplified using specific primer sequences as shown in Table 2. As an internal control to determine the possible presence of different cDNA concentrations *β-actin* was used. PCR results were visualized on a 1.6% agarose gel stained with Diamond™ Nucleic Acid Dye (Promega, Madison, WI, USA) according to the manufacturer’s instructions, visualized by Gel Imaging System (ChemiDocTM Imaging System, Bio-Rad, Hercules, CA, USA). The images were semi-quantified by an Image Lab 6.0.1 build 34 Bio-Rad Laboratories (normalized to the housekeeping gene *β-actin* mRNA levels) [39]. Results are shown as a percentage relative to the negative control of at least three biological replicates.

### 2.8. Statistical Analyses

To determine statistical significance, data were analyzed with One-way and Two-way ANOVA. Normality of data distribution was tested with Shapiro–Wilk test. Homoscedasticity of groups was tested with Bartlett’s F test. In both tests, the calculated *p* value was higher than 0.05 indicating normality of data distribution and homoscedasticity between groups, a prerequisite for ANOVA.

The main limitation of our study is the simplicity of NAS and DIS models established in Huh7 cell line. An advantage of these in vitro models is the opportunity to assess direct effect of LIRA on hepatocytes.

## 3. Results

### 3.1. Establishment of the Cell Culture Model of DIS and NAS, and Assessment of the Effect of Amiodarone and Oleic Acid on Cell Viability

**Figure 1:**
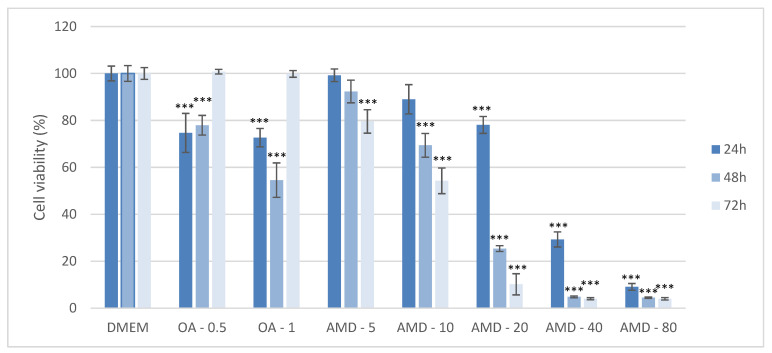
Determination of cell viability by MTT assay after exposure to varying amiodarone (AMD) and oleic acid (OA) concentrations and varying time periods in the Huh7 cell line. MTT measurements were done by spectrophotometry at 595 nm. Results are shown as a percentage relative to the negative control of at least three biological replicates. Two-way ANOVA Time period F (2, 71) = 88.8; *p* = 7.41 × 10^−17^, Treatment F (7, 71) = 741.1; *p* = 1.23 × 10^−46^; post-hoc Tukey HSD. Bars assigned with asterisks are statistically significantly different (*** *p* < 0.001) compared to the negative control. Dulbecco’s modified Eagle’s medium (DMEM), oleic acid (OA/mM), amiodarone (AMD/µM).

The toxicity of AMD and OA in Huh7 cells was assessed by MTT assay after treatment with five different AMD concentrations (5, 10, 20, 40, and 80 µM), and two different concentrations of OA (0.5 and 1 mM) at various time points: 24 h, 48 h, 72 h. Each experiment was repeated at least three times to ensure consistency of the results. After 24 h exposure to 0.5 mM OA, cells had 80% viability, the same as exposure to 20 µM AMD at the same time point. Cell viability significantly decreased to 30% in cells treated with 40 µM AMD at 24 h. The viabilities of AMD-treated cells were significantly different compared to negative DMEM control as shown in Figure 1. Exposure of cells for longer time periods (48 h, 72 h) led to a significant drop in cell viability even with lower concentrations of AMD as shown in Figure 1 (*p* < 0.001). From the MTT results, the concentrations of 0.5 mM OA, and 20 µM AMD and a duration of exposure of 24 h were selected as the Huh7 models of NAS and DIS, respectively, in all subsequent experiments.

### 3.2. Assessment of the Protective Effect of LIRA (LIRA) on Cell Viability in DIS and NAS Cell Culture Models

**Figure 2:**
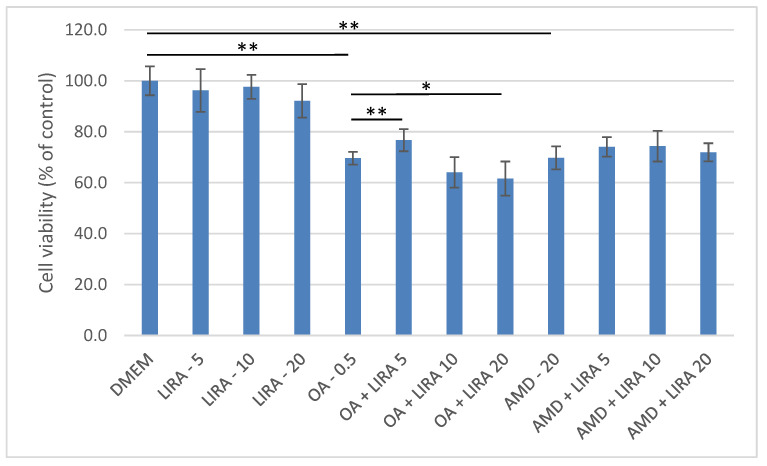
Determination of cell viability by MTT assay after exposure of Huh7 cells to OA, AMD and different LIRA concentrations for 24 h. MTT measurements were done by spectrophotometry at 595 nm. Results are shown as a percentage relative to the negative control of at least three biological replicates. One-way ANOVA F (11, 71) = 37.61; *p* = 8.38 × 10^−23^ Mann-Whitney pairwise. Bars assigned with asterisks indicate statistically significant differences (* *p* < 0.05, ** *p* < 0.01). Dulbecco’s modified Eagle’s medium (DMEM) as a negative control, liraglutide (LIRA/nM), oleic acid (OA/mM), amiodarone (AMD/µM).

Three different concentrations of LIRA (LIRA) (5, 10, and 20 nM) were used to assess its hepatoprotective effect in NAS and DIS cell culture models as shown in Figure 2. Cell viability was determined, and compared to the negative control, and to cells treated with OA and AMD. In the NAS model (cells treated with OA), 5 nM LIRA significantly increased cell viability (*p* < 0.01). On the other hand, higher LIRA concentrations had the opposite effect. All three concentrations of LIRA had a beneficial effect on cell viability in the DIS model, but without statistical significance.

### 3.3. Quantification and Visualization of Lipid Accumulation in DIS and NAS Cell Culture Models, Incubated with Varying Concentrations of LIRA

**Figure 3:**
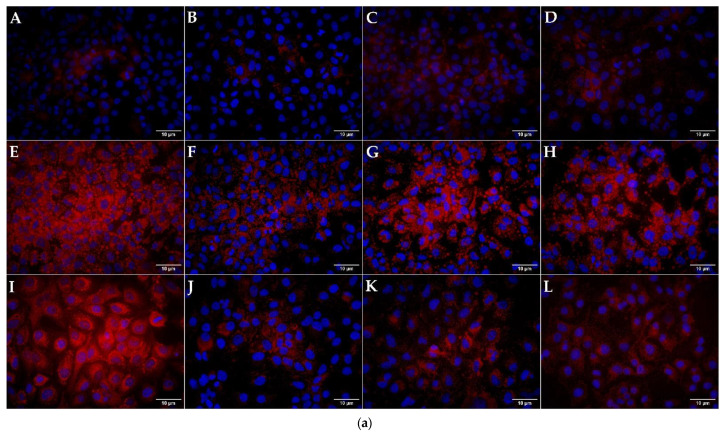

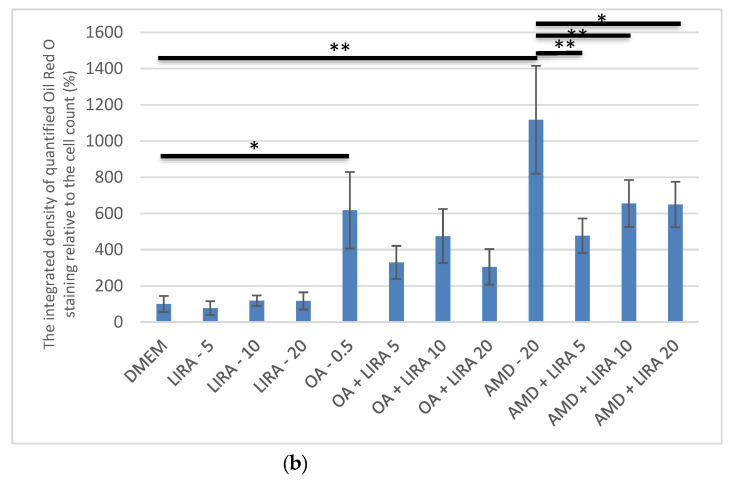
Quantification and visualization of the lipid accumulation with Oil-Red O-dye. (**a**) Visualization of lipid accumulation with Oil-Red-O dye. Lipid accumulation in Huh7 cells was visualized with Oil-Red-O dye, while DAPI (blue color) was used to stain nuclei. (**A**)—DMEM (negative control), (**B**)—5 nM LIRA, (**C**)—10 nM LIRA, (**D**)—20 nM LIRA, (**E**)—0.5 mM OA, (**F**)—0.5 mM OA and 5 nM LIRA, (**G**)—0.5 mM OA and 10 nM LIRA, (**H**)—0.5 mM OA and 20 nM LIRA, (**I**)—20 µM AMD, (**J**)—20 µM AMD and 5 nM LIRA, (**K**)—20 µM AMD and 10 nM LIRA, (**L**)—20 µM AMD and 20 nM LIRA. Size bar represents 10 µm. Dulbecco’s modified Eagle’s medium (DMEM), liraglutide (LIRA/nM), oleic acid (OA/mM), amiodarone (AMD/µM). (**b**) Levels of lipids stained with Oil-Red-O dye in the Huh7 cells treated with OA, AMD, and LIRA. Data represent the integrated density of red color relative to the cell count. A higher number equals a more intense stain. Results are shown as a percentage relative to the negative control. One way ANOVA F (11, 130) = 66.2; *p* = 2.54 × 10^−45^; Mann-Whitney U (Bonferroni-corrected *p*-value). Bars assigned with asterisks are statistically significantly different (* *p* < 0.05, ** *p* < 0.01). The data are shown as the means ± SD (standard deviation) from at least three biological replicates. Dulbecco’s modified Eagle’s medium (DMEM), liraglutide (LIRA/nM), oleic acid (OA/mM), amiodarone (AMD/µM).

Incubation of Huh7 cells with OA and AMD induced a statistically significant increase in lipid accumulation as shown in Figure 3 (*p* < 0.05, *p* < 0.01). AMD induced microsteatosis with nuclei positioned in the center of the cells, while lipid droplets in OA were larger indicating macrosteatosis occurrence. Cotreatment with LIRA reduced the lipid accumulation in both NAS and DIS models, but only the DIS model results were statistically significant (*p* < 0.01, *p* < 0.05).

### 3.4. Assessment of the Effect of LIRA on Triglyceride Accumulation in NAS and DIS Cell Culture Models

**Figure 4:**
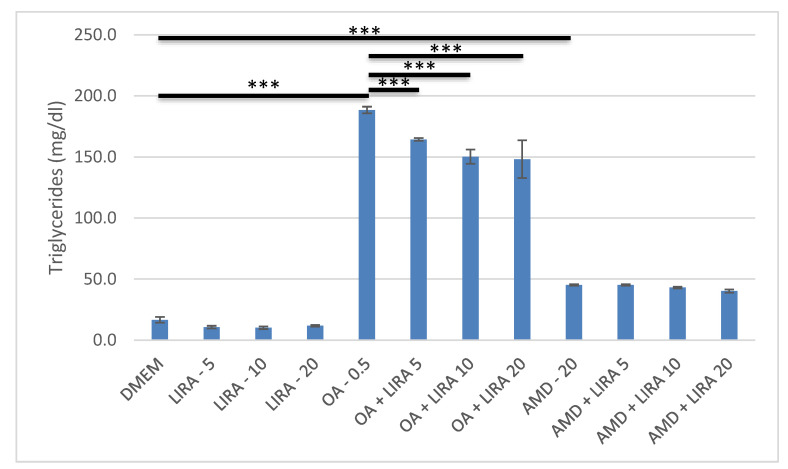
Triglyceride content in DIS and NAS cell culture models, incubated with varying concentrations of LIRA. One-way ANOVA F (11, 35) = 579.2; *p* = 2.62 × 10^−26^ post-hoc Tukey HSD. Bars assigned with asterisks are statistically significantly different (*** *p* < 0.001). The data are shown as the means ± SD (standard deviation) from at least three biological replicates. Dulbecco’s modified Eagle’s medium (DMEM), LIRA (LIRA/nM), oleic acid (OA/mM), amiodarone (AMD/µM).

AMD and OA increased significantly triglyceride content in Huh7 cells (*p* < 0.001). LIRA reduced the triglyceride accumulation in NAS model significantly, while in the DIS model, it resulted in no significant change in triglyceride accumulation, as shown in Figure 4.

### 3.5. Assessment of the Effect of LIRA on Gene Expression in NAS and DIS Cell Culture Models

**Figure 5:**
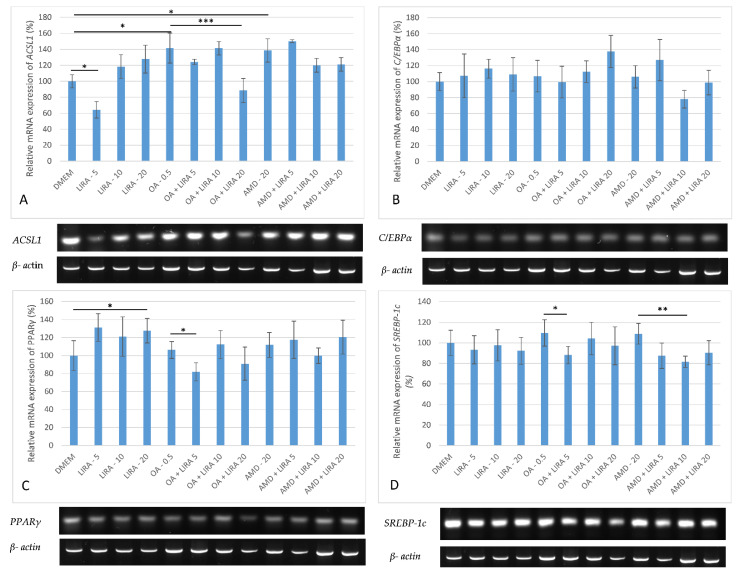
Expression of *ACSL1, C/EBPα, PPARγ* and *SREBP-1c* in cell culture models of NAS and DIS. The gene expression was determined by RT-PCR. PCR results were visualized on a 1.6% agarose gel stained with Diamond™ Nucleic Acid Dye (Promega, Madison, WI, USA) according to the manufacturer’s instructions, visualized by Gel Imaging System (ChemiDocTM Imaging System, Bio-Rad, Hercules, CA, USA). The images were semi-quantified by Image Lab 6.0.1 build 34 Bio-Rad Laboratories (normalized to the housekeeping gene *β-actin* mRNA levels) (39). Results are shown as a percentage relative to the negative control of at least three biological replicates. (**A**) *ACSL1* gene expression in Huh7 cells. One-way ANOVA F (11, 35) = 12.8 *p* = 1.63 × 10^−07^ post-hoc Tukey HSD test; * *p* < 0.05, *** *p* < 0.001. (**B**) *C/EBPα* gene expression in Huh7 cells. One-way ANOVA F (11, 47) = 2.661, *p* = 0.01317; post-hoc Tukey HSD test. (**C**) *PPARγ* gene expression in Huh7 cells. One-way ANOVA F (11, 59) = 4.415, *p* = 0.000142; Mann-Whitney U test * *p* < 0.05. (**D**) *SREBP-1c* gene expression in Huh7 cells. One-way ANOVA F (11, 59) = 4.241, *p* = 0.000212; post-hoc Tukey HSD test; * *p* < 0.05, ** *p* < 0.01. Dulbecco’s modified Eagle’s medium (DMEM), LIRA (LIRA/nM), oleic acid (OA/mM), amiodarone (AMD/µM), Acyl-CoA Synthetase Long Chain Family Member 1 (*ACSL1*), CCAAT/enhancer-binding protein α (*C/EBPα*), peroxisome proliferator-activated receptor gamma (*PPARγ*), sterol regulatory element binding transcription factor 1c (*SREBP-1c*).

To determine the effect of LIRA in NAS and DIS models on the mRNA levels of *ACSL1*, *C/EBPα*, *PPARγ*, and *SREBP-1c* RT-PCT was performed. The mRNA levels in the gels were representative results of at least three biological replicates, and the values reported as percentages relative to the negative control. Gene expression was quantified by Image Lab 6.0.1 build 34 Bio-Rad Laboratories, and normalized to the housekeeping gene *β-actin* mRNA levels. Because the densities of blot images are frequently non-uniform, visual impressions are not as accurate as the data from the image analyzer. Both OA and AMD upregulated *ACSL1* gene expression (*p* < 0.05), while LIRA in cotreatment decreased *ACSL1* levels, but only the OA NAS model results were statistically significant (*** *p* < 0.001), as shown in Figure 5A. *C/EBPα* was downregulated with lowest LIRA treatment in NAS model, while in the DIS model higher LIRA concentrations achieved downregulation, all without statistical significance, as shown in Figure 5B. The effect of LIRA on *PPARγ* gene expression is shown in Figure 5C. OA increased the mRNA levels of *PPARγ*, while 5 nM and 20 nM LIRA reversed this effect (*p* < 0.05). In the AMD DIS model, *PPARγ* gene expression was upregulated and 10 nM LIRA reversed this effect, but without statistical significance. In both models, a slight increase in *SREBP-1c* expression was observed, whereas 5 nM LIRA significantly downregulated its expression in NAS model (*p* < 0.05), and 10 nM LIRA in DIS model (*p* < 0.01), as shown in Figure 5D.

## 4. Discussion

So far, various in vitro models for NAS have been established, mostly with palmitic acid, and/or OA added to different cell cultures. In our study, we used OA and a Huh7 cell line. Although Ricchi et al. demonstrated that OA had no effect on cell viability, our research showed a significant decrease in cell viability (20%) after treatment of Huh7 cells with 0.5 mM OA for 24 h and 48 h [35]. This effect was no longer present after 72 h of treatment. A possible explanation could be that cells had time to recover, and OA was added only at the beginning of this experiment.

After an extensive literature search, AMD was selected as a key compound for the induction of DIS. It has been shown to have a hepatosteatotic effect and is frequently used in clinical practice. Unlike some other drugs that induce hepatosteatosis, amiodarone has not been shown to have an extrahepatic effect that could significantly contribute to its hepatosteatotic activity. Therefore, AMD exerts hepatosteatotic effect by direct effect on hepatic cells, making it suitable for cell culture study [42]. After treating cells with various AMD concentrations at different time periods, a concentration of 20 µM and an incubation time of 24 h were selected because of the significant effects on cell viability, without causing apoptosis to a greater extent (that occurs with higher amiodarone concentrations, and longer incubation times). The same treatment conditions have already been used in previous studies (9). Comparing the NAS and DIS models, the effects on cell viability were similar, but OA induced triglyceride accumulation to a greater extent compared to AMD, while AMD had a greater effect on lipid accumulation/cell in general, as shown with Oil-Red-O staining. Zhou et al. showed a significant, three-fold, increase in the lipid droplet number per cell in HepG2 cells treated with AMD for 24 h [42,43]. In the current study, an even greater-, tenfold, increase in the integrated density of quantified Oil-Red-O staining relative to cell number was observed compared to the negative control. Vitins et al. demonstrated the triglyceride accumulation as one of the main characteristics in AMD DIS, but only with longer incubation periods. However, after 24 h treatment, only a slight increase was observed [8]. In our current study, triglycerides were increased three-fold. However, considering the much greater increase in lipid accumulation as shown by Oil-Red-O staining, it is possible that accumulation of other lipids in the liver due to AMD occurred as well. Accordingly, in one in vivo study, accumulation of phosphatidylcholine occurred after AMD treatment [8,44]. On the contrary, triglyceride accumulation was significantly increased in OA NAS model as has been reported in previous studies [35].

The histological pattern also differed between these two models. Amiodarone DIS caused a microsteatosis which is consistent with the findings of previous studies [6,9,10,35], while NAS resulted in larger vesicles (macrosteatosis). NAS is usually associated with macrovesicular steatosis, although Tandra et al. demonstrated that microvesicular steatosis can also be present in approximately 10% of biopsies from patients with NAS [45,46]. Both models, NAS and DIS, showed an increase in *ACSL1* (protein that converts free long-chain fatty acids into fatty acyl-CoA esters) gene expression. This increase has been demonstrated in several previous studies of NAS models, but from our literature search, this is the first report of an AMD-induced increase in *ACSL1* [27]. Accordingly, the increased triglyceride accumulation detected in our models is, at least partly, due to the upregulation of *ACSL1* which has been reported to be involved in both the reduction of fatty acid β-oxidation and the induction of lipogenesis in the liver [47,48]. Li at al. demonstrated that this effect was achieved through the *PPARγ* signaling pathway [47]. Both models, NAS and DIS, also showed significant upregulation of *PPARγ* expression. Previous studies have also demonstrated an increase in liver *PPARγ* expression in NAS model [13,49,50]. This may indicate a possible steatogenic role of *PPARγ* in the liver in triglyceride accumulation and lipid droplet formation in both models of fatty liver [51,52]. A slight increase in *C/EBPα* and *SREBP-1c* gene expression was observed in the NAS and DIS models, confirming their role in enhancing fatty changes [27,53].

LIRA concentrations were selected after evaluating other studies, which reported that the maximum clinical dose administered to patients was 3 mg of liraglutide daily. Extension of this figure to a person of 85 kg of weight resulted in a dose of 11.3 nmol/kg. We used 5 nmol/L, 10 nmol/L and 20 nmol/L of LIRA. Accordingly, we chose a mass to volume ratio of water of around 1. It is possible that the selected doses of LIRA used in the current study might have had a hypoglycemic effect when applied to cells. However, LIRA increases insulin secretion, while inhibiting glucagon, only in response to increases in glucose levels [54]. Therefore, the risk of hypoglycemia should be negligible.

In the current model, concentrations higher than 20 nM significantly reduced cell viability (although some studies used even 100 nM and 500 nM), so we decided to use 5, 10, and 20 nM LIRA for the experiments [16,17]. Although the lowest LIRA concentration used in our study, 5 nM, had a slightly positive effect on cell viability, higher concentrations decreased cell viability. However, these concentrations were 2-fold higher than those used in clinical practice. Consistent with other studies, LIRA generally reduced lipid accumulation in NAS model, and exerted an even stronger effect in DIS model [24,55]. The authors found no similar study demonstrating antisteatotic effect of LIRA in DIS model. However, in the current NAS model, LIRA also reduced lipid droplet size, suggesting an inverse effect on macrosteatosis which was supported by a significant decrease in triglyceride content (triglycerides are thought to be the main contributor to lipid droplet enlargement) [56,57]. When applied to the cells treated only with low-glucose DMEM medium, LIRA downregulated *ACSL1* gene expression in 5 nM, but increased it in 10 nM and 20 nM groups. These results were unexpected, but reproducible, and we have no data to explain them. It is possible that high-affinity binding to *ACSL1* transcription factors or their regulators may have decreased *ACSL1* activity while low-affinity binding had the opposite effect. Further research is necessary in order to better understand underlying mechanisms. With the exception of 5 nM LIRA in the DIS model, all other concentrations of LIRA resulted in the downregulation of *ACSL1* in both models, suggesting that LIRA achieved its antisteatotic effects in part by affecting the *ACSL1* signaling pathway and reducing triglyceride synthesis. Flock et al. demonstrated a similar effect for another drug that enhances the GLP-1R effects, vildagliptin [58].

LIRA upregulated *PPARγ* in cells incubated only in a low-glucose medium, similar to previous in vivo studies, suggesting that it is responsible for balancing the lipid metabolism in hepatocytes [13]. Decara et al. demonstrated that in lean rats, LIRA upregulated genes involved in lipogenesis including *PPARγ*, whereas in high-fat-diet-induced obesity rats, the decrease in lipid accumulation by LIRA occurred due to a decreased expression of *PPARγ* in liver [13]. Accordingly, when added to NAS and DIS models in our study, LIRA mainly reduced *PPARγ* expression. It is also interesting to note that this effect was not dose-dependent, and some concentrations of LIRA even had the opposite effect. This result could be explained by the diverse role of *PPARγ* in the liver. Various studies demonstrated an antifibrotic role of *PPARγ* activation in the liver [59,60,61]. Ni et al. and Liu et al. showed that *PPARγ* is necessary for the inactivation of human hepatic stellate cells (HSCs), and regression of liver fibrosis in mice [59,60]. Additionally, *PPARγ* inhibits HSCs proliferation, and decreases extracellular-matrix production by inhibition of the activation of the TGF-β1/Smad signaling pathway [51,59,62]. Finally, *PPARγ* agonism enhanced sensitivity of adipose tissue, muscle, and liver to insulin, and this effect probably overcame negative effects of *PPARγ* on fat accumulation in hepatocytes, and resulted in a decrease in fat. Decara et al. showed that LIRA slightly upregulated *PPARy* in adipose tissue of HFD-induced obesity rats [13,26].

The effect of LIRA on *C/EBPα* gene expression was variable in our study, and without statistical significance. LIRA cotreatment had a different effect in NAS and DIS models, with a greater reduction in *C/EBPα* mRNA levels in the DIS model. Guzman et al. demonstrated that *C/EBPα* represses liver fatty acid binding protein (*FABP1*) responsible for the prevention of lipotoxicity of FFAs ad regulation of FFA trafficking and partition [53]. *C/EBPα* was induced or did not change in human NAS or animal models of NAS [53]. However, various studies showed also an antifibrotic role of *C/EBPα* in the liver achieved by induction of HSCs apoptosis [63,64,65,66]. To date, authors have not studied LIRA effects on *C/EBPα* expression in the liver, although various studies confirmed its downregulating effect in adipose tissue during longer treatment periods [67,68]. More studies are necessary in order to evaluate the role of *C/EBPα* in liver.

*SREBP-1c* gene expression was significantly downregulated with LIRA cotreatment in both models in the current study which confirms the role of *SREBP-1c* gene pathway in LIRA antisteatotic effect. That is consistent with the findings of Wang et al. who demonstrated the effect of LIRA in decreasing lipid content in liver by the *AMPK/SREBP1* pathway [24]. *AMPK* regulates the long-term adaptation of lipid metabolism in liver by downregulation of *SREBP-1c*. Therefore, upregulation of *SREBP-1c* leads to disturbance in lipid metabolism and accumulation of fat in liver [24]. In the current study, gene expression could have been confirmed by protein analyses, in situ hybridization, and confocal microscopy studies, but the issues to be addressed by those methods are beyond the scope of this report.

## 5. Conclusions

In conclusion, LIRA has been shown to have hepatoprotective and antisteatotic effects in NAS and DIS cell culture models established by incubating the Huh7 cell line with OA and AMD, respectively. This effect was achieved by downregulation of gene expression of several elements involved in lipid accumulation (*ACSL1*, *C/EBPα*, *PPARγ*, *SREBP-1c*). However, the role of some of these gene pathways in the liver has not been fully elucidated. Overall, our studies suggest that LIRA may play an important role in the treatment of not only NAS, but also DIS. However, further studies are needed to clarify the exact role of the various gene pathways in the development of fatty liver, not only in hepatocytes, but also in other liver cells in order to confirm the hepatoprotective role of LIRA in various fatty liver models.

## Figures and Tables

**Table 1:** PCR conditions for amplification of different genes.

Gene	Denaturation	Annealing	Elongation
*β-actin*	94 °C for 3 min	56.7 °C for 45 s	72 °C for 1 min in 30 cycles
*ACSL1*	94 °C for 3 min	61 °C for 45 s	72 °C for 1 min in 30 cycles
*C/EBPα*	94 °C for 3 min	61 °C for 45 s	72 °C for 1 min in 30 cycles
*PPARγ*	94 °C for 3 min	61 °C for 45 s	72 °C for 1 min in 30 cycles
*SREBP-1c*	94 °C for 3 min	61 °C for 45 s	72 °C for 1 min in 30 cycles

**Table 2:** Primer sequences used for RT (Reverse transcription)-PCR.

Gene	Primer Sequences (5′-3′)
*β-actin*	Forward GCACCACACCTTCTACAATG Reverse TGCTTGCTGATCCACATCTG
*ACSL1*	Forward GGAGTGGGCTGCAGTGAC Reverse GGGCTTGCATTGTCCTGT
*C/EBPα*	Forward CGCCTTCAACGACGAGTTCCTG Reverse CGCCTTGGCCTTCTCCTGCT
*PPARγ*	Forward ACCAAAGTGCAATCAAAGTGGA Reverse ATGAGGGAGTTGGAAGGCTCT
*SREBP-1c*	Forward CGGAACCATCTTGGCAACAGT Reverse CGCTTCTCAATGGCGTTGT

*ACSL1*—Acyl-CoA Synthetase Long Chain Family Member 1, *C/EBPα*—CCAAT/enhancer-binding protein α, *PPAR γ*—peroxisome proliferator-activated receptor gamma, *SREBP-1c*—sterol regulatory element binding transcription factor 1c [40,41].

## Data Availability

The data presented in this study are available on request from the corresponding authors.
